# A Review of Community Health Worker Integration in Health Departments

**DOI:** 10.1007/s10900-023-01286-6

**Published:** 2023-10-12

**Authors:** Stacy Ignoffo, Shannon Gu, Alexander Ellyin, Maureen R. Benjamins

**Affiliations:** 1 Sinai Urban Health Institute, Chicago, IL USA; 2 Chicago Medical School, Rosalind Franklin University of Medicine and Science, North Chicago, IL USA

**Keywords:** Community health workers, Promotoras de salud, Health departments, Certification, Public health, Health inequities

## Abstract

Community health workers (CHWs) are frontline public health workers who bridge the gap between historically marginalized communities, healthcare, and social services. Increasingly, states are developing the CHW workforce by implementing training and certification policies. Health departments (HDs) are primarily responsible for community health through policy implementation and provision of public health services. The two objectives of this study are to explore: (1) state progress in establishing CHW training and certification policies, and (2) integration of CHWs in HD workforces. In this scoping review, we searched PubMed, CINAHL, and Google Scholar for articles published between 2012 and 2022. We looked for articles that discussed state-level certification and training for CHWs and those covering CHWs working with and for city, county, state, and federal HDs. We excluded studies set outside of the US or published in a language other than English. Twenty-nine studies were included for review, documenting CHWs working at all levels of HDs. Within the included studies, HDs often partner with organizations that employ CHWs. With HD-sponsored programs, CHWs increased preventative care, decreased healthcare costs, and decreased disease risk in their communities. Almost all states have begun developing CHW training and certification policies and are at various points in the implementation. HD-sponsored CHW programs improved the health of marginalized communities, whether CHWs were employed directly by HDs or by a partner organization. The success of HD-sponsored CHW programs and state efforts around CHW training and certification should encourage increased investment in CHW workforce development within public health.

## Introduction

As trusted members of the communities they serve, community health workers (CHWs) are valuable assets to the healthcare, social service, and public health workforce. CHWs bridge the gap, serving as connectors between their communities, the healthcare system, and social services, particularly in historically marginalized and/or underserved populations. CHW-led interventions have been shown to reduce healthcare utilization, lower the risk of cardiovascular disease, promote cancer screening, and improve diabetes outcomes, among other benefits [1–3]. CHWs help community members navigate health care and other systems, and address health-related social needs by referring community members to resources for food, transportation, housing, and other social services [4, 5].

CHWs work in a variety of organizations, with over 80% employed by non-profit organizations, community clinics, or hospitals [6]. Fewer than 10% of CHWs work for health departments [6]. Health departments, as governmental agencies, coordinate and provide a range of public health services at local, state, and federal levels. Their services span domains such as communicable diseases, environmental health, immunizations, and community outreach. Health departments are also heavily involved in training and certifying public health professionals, including CHWs, although their involvement varies state to state. While the overall mission of most health departments is to improve community health, in recent years, an increasing number of health departments, from the federal level down, are also focusing on racial health inequities [7].

The number of CHWs working within, or in partnership with, health departments has increased dramatically in recent years. The acceleration of state policies for certification and reimbursement of CHWs has been a major driving factor. In addition, since 2020, health departments have employed more CHWs as part of their COVID-19 pandemic response efforts [8]. For instance, the Illinois Department of Public Health used pandemic relief funds from the federal government to establish the Pandemic Health Navigator Program, a workforce of over 650 CHWs [8]. With the end of the public health emergency, it is important to understand how CHWs are integrated more broadly into the work of health departments. To this end, the current study explores how health departments in the United States: (1) support the CHW workforce through CHW training and certification policies, and (2) integrate CHWs into their workforce.

## Methods

In this review, we used a scoping review methodology to assess the scope of CHW research as it relates to health departments, drawing from both peer-reviewed literature and gray literature. From July 2022 to December 2022, we searched PubMed, CINAHL, and Google Scholar for peer-reviewed literature published between 2012 and 2022. We included articles that discussed CHWs along with health departments at any level, including city, county, state, and federal. Our search strings included combinations of the following terms: “community health worker”, “[state name]”, “health department” OR “department”, “training”, and “certification”. We excluded articles that were set outside of the United States, were published in a language other than English, or did not mention health departments. After screening abstracts and full text, we compiled key characteristics from each study into a standardized table, including elements such as study design, CHW program interventions, and quantitative and qualitative outcomes. We also searched gray literature for news articles and press releases about the role of CHWs during the COVID-19 pandemic.

## Results

Twenty-nine studies were selected for inclusion in this review. Table 1 reflects “health department sponsored programs” which includes programs where CHWs are employed by the health department, and programs where CHWs are employed by another organization but collaborate with a health department. Table 2 reflects studies related to CHW training and certification. The earliest studies were published in 2013, and 14 of the studies were published in 2020 or later. Twelve studies focused on CHW training and certification, while the remaining 17 studies focused on CHW programs associated with health departments. Study designs varied from case studies to randomized controlled trials, and study results included both quantitative and qualitative data.

**Table 1:** Studies about health department-sponsored CHW programs

Study	Health department	Health department level	CHW program description	CHW roles	Results
Blewett and Owen (2015)	Hennepin County Human Services and Public Health Department	County	Hennepin Health was an accountable care organization that provided medical and social services to low-income Medicaid beneficiaries in Hennepin County. CHWs were employed at participating clinics under Hennepin Health	Coordinated health and social services	Decreased ED visits by 9% from 2012 to 2013 Decreased inpatient admissions by 3% from 2012 to 2013
Brown et al. (2018)	City of El Paso Department of Public Health	City	Healthy Fit aimed to address Hispanic health inequities in cardiovascular disease and preventative health access. Participants included uninsured individuals or Medicaid beneficiaries 18 years or older	Conducted health screenings and provided vouchers for cancer screenings and vaccinations Conducted follow-up interviews to assess participant follow-through on referrals	54% of breast cancer screening referrals, 43% of cervical cancer screening referrals, and 32% of colorectal cancer screening referrals were completed
Buder et al. (2018)	Utah Department of Health	State	The Coalition for a Healthier Community for Utah Women and Girls, University of Utah, Utah Department of Health, and racial and ethnic community leaders designed a CHW program to improve diet and physical activity among minority women	Motivational interviewing to assess diet and exercise goals	Significantly more participants were physically active and eating adequate daily servings of fruits and vegetables after 1 year (p < 0.001)
Chernoff and Cueva (2017)	U.S. Department of Health and Human Services	Federal	Alaska's Community Health Aides/Practitioners (CHA/Ps) are healthcare providers who work in rural and remote areas of Alaska. Their scope of practice is defined by the Indian Health Service under the U.S. Department of Health and Human Services	Provided prenatal care, patient education about pregnancy, emergency delivery services, and well-child visits Provided education to teenagers about tobacco, alcohol, depression, and sex education	CHA/Ps often provide the only access to healthcare in rural and remote Alaskan communities
Cooper et al. (2021)	Richmond City Health District, Virginia Department of Health	City and State	The Virginia Department of health implemented resource centers in public housing. The resource centers were staffed by CHWs and nurses, who focused on healthcare access and preventative care	Educated residents about preventive care and chronic disease intervention Referred residents to community resources for health and social needs	Educated 2609 public housing residents Made 9591 referrals to health, social, and community services
DeAngelis et al. (2017)	U.S. Department of Health and Human Services	Federal	Healthy Start is a nationwide program to improve birth outcomes, prevent infant mortality, and reduce inequities in maternal and infant health	Provided health education and coaching Referred participants to social services	91% of Healthy Start programs employed CHWs CHW training varied widely across programs
Gratale and Haushalter (2016)	Delaware Public Health Department	State	Nemours Children’s Health System partnered with community organizations to coordinate community-based prevention and clinical care for children with asthma. CHWs recruited participants and worked with children and their families, Nemours care coordinators, and other community organizations	Helped families identify and mitigate asthma triggers in their homes and other environments Connected families with services such as food, housing, and childcare	Decreased asthma-related ED visits by 60% from 2012 to 2014 Decreased healthcare costs by $533 per child per quarter relative to comparison group
Hammack et al (2021)	Louisiana Department of Health	State	The Ending the HIV Epidemic in the U.S. Initiative funded a CHW program in East Baton Rouge Parish, Louisiana to reduce the incidence of HIV and support people with HIV	Conducted HIV testing and assessed need for HIV medical care and pre-exposure prophylaxis	Screened 320 community members for HIV, connected 10 to HIV medical care, and helped 19 with pre-exposure prophylaxis
Heisler et al. (2022)	Detroit Health Department	City	Medicaid health plans, the Detroit Health Department, a community organization, and a university implemented a CHW program in a low-income neighborhood in Detroit. CHWs provided services to Medicaid beneficiaries in the neighborhood	Conducted health, behavioral, and social needs assessments Developed individualized action plans for beneficiaries Connected beneficiaries to social services	Participants in the CHW program had fewer ED visits (ARR = 0.96; 95% CI =0 .94, 0.98; p < 0.01) and ED visit costs (ARR = 0.96; 95% CI = 0.94, 0.98; P < 0.001)
Kaufman et al (2014)	U.S. Department of Veterans Affairs	Federal	Tribal Veterans Representatives (TVRs) are volunteer outreach workers who serve as liasons between American Indian and Alaska Native veterans and the Department of Veterans Affairs (VA). The TVR program aimed to connect American Indian and Alaska Native veterans in rural communities to VA benefits and services	Conducted outreach to American Indian and Alaska Native veterans at their homes and at community events Connected American Indian and Alaska Native veterans to the VA's healthcare services	Increased American Indian and Alaska Native veterans' healthcare access through establishing a primary care clinic, a transportation system to hospitals, and a resource center
Krantz et al. (2013)	Colorado Department of Public Health and Environment	State	Colorado Heart Healthy Solutions was a CHW-run program that aimed to prevent coronary heart disease across 34 Colorado counties	Performed health screenings including blood pressure, weight, blood glucose, and cholesterol Motivational interviewing Followed up on participants’ referrals	Significant decreases in weight, blood pressure, cholesterol, and heart disease risk after 3 + months
Lohr et al. (2021)	Arizona county health departments	County	CHWs in Latino populations along the U.S.-Mexico border worked to support participants' needs and well-being	Met with participants monthly for 6 months Taught stress management and relaxation skills	Increase in participants' perceived social support, hopefulness, and quality of life (p < 0.001)
Obasanjo et al. (2022)	Virginia Department of Health	State	CHWs worked in low-income housing resource centers in the Richmond/Henrico Health District	Referred residents to community resources Measured blood pressures and blood glucose	Made 17,580 total referrals to resources including healthcare access (49%) and economic stability (38%)
Portillo, Vasquez and Brown (2020)	City of El Paso Department of Public Health	City	Healthy Fit aimed to reduce the risk of chronic diseases in El Paso's Hispanic immigrant community. CHWs recruited and screened participants at the Mexican consulate's Ventanilla de Salud, community events, and health fairs. Participants included uninsured individuals or Medicaid beneficiaries 18 years or older	Conducted health screenings Provided vouchers for health resources Motivational interviewing	Over 2,500 participants received vouchers for cancer screenings and immunizations, alcohol and tobacco education, or resources on healthy lifestyle changes
Sabo et al. (2021)	Arizona Department of Health Services	State	The Arizona Health Start Program was a CHW home visiting program that aimed to improve maternal and child health outcomes among high risk mothers with children ages 0–2 years	Provided perinatal and postpartum education Screened for maternal behavioral health disorders, alcohol and tobacco use, and intimate partner violence	Program participation was associated with a 38% lower risk of low birth weight in American Indian mothers, a 36% lower risk of very low birth weight in Latina mothers, and a 30% lower risk of preterm birth in teenage mothers
Stupplebeen et al. (2019)	Hawaii Department of Pubilc Health	State	The Hawaii Department of Public Health, Hawaii Primary Care Association, and federally qualified health centers engaged CHWs to address inequities in diabetes and hypertension among Native Hawaiians, Pacific Islander, and Filipino populations	Connected participants to resources such as food banks, behavioral health providers, and wellness classes Implemented intervention activities such as self-measured blood pressure monitoring programs	CHWs build networks by connecting with community resources or creating their own resources CHWs face barriers and unmet needs in developing community-clinical linkages
Turner et al. (2020)	Texas Department of Health and Human Services	State	The Texas Transformation and Quality Improvement Program piloted a CHW program to improve diabetes and hypertension control among low-income Hispanic primary care patients	Contacted participants by telephone and/or in clinic Educated patients about diabetes medications and devices and lifestyle modifications	Patients who saw CHWs in clinic achieved blood glucose control more rapidly than patients who were only contacted via telephone (HR 1.45, p = 0.043)

**Table 2:** Studies related to CHW training and certification

Study	Location	Findings
Allen et al. (2015)	Nationwide	State health departments can more effectively integrate CHWs into healthcare by defining their scope of practice, developing and sustainably funding the workforce, building statewide support, and engaging them in community-clinical linkages
Barbero et al. (2021)	Nationwide	(1) 12 strategies in implementing statewide CHW workforce development initiatives were identified. On average, states addressed 8 of the strategies in their initiatives (2) 85% of states implemented the following strategies: leveraging investments in CHW initiatives; establishing statewide definitions, standards, and/or policy for CHW workforce; and creating statewide training and development opportunities for CHWs (3) Fewer than 25% of states implemented the following strategies: assessing statewide employer readiness and support for CHWs and assessing increased statewide CHW employment (4) As of September 2020, 45 states had a multi-stakeholder CHW coalition and 31 states had a statewide CHW organization
Dunklee and Garneau (2018)	Rhode Island	(1) The Rhode Island Department of Health developed CHW certification standards in 2016. 217 CHWs were certified as of 2018 (2) Rhode Island CHWs are mostly funded through philanthropic or public grant funding. Funding goes towards CHW workforce development, CHW trainers, and CHW employers
Dunn et al. (2021)	Texas	(1) 9% of CHW employers surveyed were local health departments and 3% were state agencies (2) 75% of CHW employers required CHW certification from the Texas Department of State Health Services (3) Key CHW workforce development needs included continued CHW training (56%) and financial support (55%). 4) CHW employers had the least capacity for compensation for certified continuing education for CHWs (39%) and development of career pathways for CHWs (39%)
Ingram et al. (2020)	Arizona	The Arizona Department of Health Services, the Arizona CHW Association, promotoras de salud, and tribal community health representatives unified to form the Arizona Community Health Worker Workforce Coalition. The coalition developed core competency training and advocated for CHW legislation. CHW voluntary certification legislation was signed into law in 2018
Rodriguez et al. (2022)	Indiana	(1) 15% of CHWs surveyed were employed at county health departments (2) 81% of CHWs surveyed were currently certified, and 94% had their certification paid for by their employer. 57% found employment as a result of certification (3) Certification programs varied widely. Not all programs were recognized by the certifying state agency or approved for reimbursement
Simonsen et al. (2017)	Utah	The first statewide CHW coalition (Utah CHW Coalition) was formed in 2015. The coalition focused on sustainable reimbursement, standardized training, certification, defined scope of practice, and public and professional recognition for the work of CHWs
Sugarman et al. (2021)	Louisiana	(1) 11% of CHWs surveyed were employed at health departments (2) CHW-identified benefits of certifcation included the opportunity to learn new skills (82%), improved job opportunities (80%), and clearly defined roles and expectations (79%) (3) CHW-identified concerns about certification included not wanting non-CHWs to create standards for certification (44%), not having the resources to pay for certification (42%), and already having the necessary skills based on their experience (32%) (4) CHW employer-identified benefits of certifcation included helping CHWs learn new skills (82%), improving their work performance (76%), and expanding the workforce (73%) (5) CHW employer-identified concerns about certification included changing the way community members perceive CHWs (42%) and CHWs not having the resources to pay for certification (32%)
Trout et al. (2020)	Nebraska	(1) 32% of CHW employers surveyed were local health departments (2) A majority of organizations encouraged continuing education and offered group training sessions with other organizations for CHWs (3) 67% of CHW employers felt they needed to hire more CHWs (4) More urban than rural organizations provided education to CHWs after the CHWs began working at their organizations
Tucker et al. (2017)	Florida	(1) 22% of CHWs surveyed were employed at county health departments (2) More than half were interested in becoming certified, while fewer than one quarter were not interested (3) Most uncertified CHWs were interested in participating in training to develop core competencies and become certified
Wiggins et al. (2013)	Multnomah County, Oregon	(1) The Community Capacitation Center (CCC) at the Multnomah County Health Department has trained over 620 CHWs since 1998 (2) The CCC has also supported other organizations in the recruitment and hiring of CHWs, development of CHW job descriptions, and support and supervision of CHWs (3) The CCC participated with the Oregon Health Authority Office of Equity and Inclusion and other stakeholders to develop standards for CHW certification and approval of CHW training programs (4) 95% of CHWs felt their participation in CCC's training program improved their ability to promote health in their communities
Wilkinson et al. (2016)	Massachusetts	The Massachusetts Association of Community Health Workers and Massachusetts Department of Public Health successfully advocated for legislation authorizing CHW certification in the state. The law outlined core competencies and created a state board for certification composed of at least 4 CHWs. Certification is voluntary under the law

### Statewide CHW Workforce Development

Barbero et al. developed a model to assess progress in developing CHW initiatives in all 50 states [9]. They identified 12 strategies being used to support CHW initiatives, such as involving CHWs in statewide health systems and supporting statewide CHW organizations. On average, states have addressed eight of the 12 strategies in their initiatives. Over 46 states are investing in initiatives, establishing statewide standards and policies, and developing statewide training and development opportunities for CHWs.

As of 2021, 45 states had a multi-stakeholder CHW coalition and 31 had a statewide CHW organization [9]. For example, the Utah Department of Health established a statewide CHW coalition in 2015 focusing on sustainable reimbursement of CHWs, standardized training and certification, and a defined scope of practice for CHWs [10]. The Massachusetts Department of Public Health and Massachusetts Association of Community Health Workers were instrumental in creating CHW certification in that state [11].

Several statewide CHW census surveys have examined state-level CHW demographics and workforce development needs. The proportion of CHWs working under local health departments in each state varies widely, ranging from 9% in Texas to 22% in Florida to 32% in Nebraska [12–14].

Most surveys identified certification as a primary workforce development issue [12, 13, 15, 16]. In Louisiana, CHWs and CHW employers agreed that certification would support CHWs in learning new skills, improving job prospects, and expanding the CHW workforce [15]. Certification is not without its concerns, however. Louisiana CHWs and CHW employers were concerned that CHWs may not be able to afford certification, and most Texas CHW employers felt they did not have the capacity to compensate for certified continuing education for their CHWs [12, 15].

Progress in establishing CHW certification standards varies from state to state. Twenty-three states have certification processes in place, whether through health departments or state CHW organizations, while 14 states are in the process of developing certification standards [17]. For example, Texas already has certification processes in place, with 75% of CHW employers requiring their CHWs to be certified by the Texas Department of State Health Services [12]. Oregon is another example of state CHW certification, with standards developed by the Oregon Health Authority Office of Equity and Inclusion and the Multnomah County Health Department [18]. The Rhode Island Department of Health also created standards for certification of CHWs throughout the state [19]. In Arizona, *promotoras de salud* and community health representatives collaborated to create a voluntary certification legislative effort with support from the Arizona Department of Health Services [20]. Meanwhile, Florida and Louisiana do not have certification processes, but a majority of CHWs there have expressed interest in becoming certified [13, 15].

Other workforce development needs included funding and reimbursement of CHW services and continuing education and training for CHWs [12, 16]. In 2022, 29 out of 48 states surveyed allowed Medicaid payment for CHW services, indicating an opportunity for growth in state-level support of CHWs [21]. State health departments can also support CHW workforce development by helping to integrate CHWs in ambulatory care settings [22].

### Health Department-Sponsored CHW Programs

#### City Health Departments

There are several examples of CHW programs with city health departments. For example, the Detroit Health Department collaborated with a community organization, a university, and Medicaid health plans to create a CHW program in one of Detroit’s low-income neighborhoods [23]. The program contributed to fewer emergency department (ED) visits and lower ED visit costs [23]. In Texas, the City of El Paso Department of Public Health partnered with a university to create a CHW program called Healthy Fit, which aimed to reduce Hispanic health inequities [24, 25]. CHWs screened participants, provided vouchers for health resources, and conducted motivational interviews to further support participants [25]. Healthy Fit ultimately helped increase cancer screenings and immunizations in the community, recruiting over 2500 participants [25].

City health departments also partner with state health departments to provide CHW services. In Virginia, the Richmond City Health District and the Virginia Department of Health implemented satellite clinics staffed by CHWs and public health nurses in low-income public housing [26, 27]. The CHWs provided health screenings and referrals to other community resources, such as employment assistance.

During the COVID-19 pandemic, some city health departments further expanded their CHW workforce. In September 2021, the New York City (NYC) Department of Health and Mental Hygiene and NYC Health + Hospitals spearheaded the NYC Public Health Corps, which worked to support NYC’s public health workforce and address health inequities exacerbated by the pandemic [28]. The NYC Public Health Corps trained new CHWs and provided grants for CHWs to serve the communities most adversely affected during the pandemic. The CHWs not only provided COVID-19 preventative care, vaccinations, and treatment, but they also addressed communities’ social needs such as food and housing [28].

#### County Health Departments

There are fewer examples of CHW programs under county health departments. In Minnesota, the Hennepin County Human Services and Public Health Department and community organizations contracted with the Minnesota Department of Human Services to create an accountable care organization (ACO) [29]. The CHWs at participating clinics coordinated social services for low-income residents, and preliminary results showed the ACO decreased the rate of ED and inpatient admissions in Hennepin County [29]. Another study showed that CHWs employed by Arizona county health departments and clinics were associated with a positive impact on the emotional well-being of Latino/Latina participants, including improved social support, hopefulness, and quality of life [30].

#### State Health Departments

Most CHW programs associated with health departments were found at the state level. Many state health department sponsored CHW programs have successfully improved health outcomes, such as decreasing rates of low birth weights among minority women in Arizona, improving diet and exercise among women in Utah, and reducing the risk of coronary heart disease in counties in Colorado [31–33]. CHWs employed by state health departments have also made positive impacts on preventative care, such as in Louisiana, where CHWs helped 40% of their referred clients complete sexually transmitted infection testing and receive health education materials [34].

Additionally, state health department sponsored CHW programs have shown economic benefits. The Delaware Public Health Department partnered with Nemours Children’s Health System to promote advancement of CHWs in a collaboration between community-based prevention efforts and clinical care for children with asthma [35]. Preliminary data suggested that ED visits decreased by 60% from 2012 to 2014 and healthcare costs for children with asthma decreased compared to children in the control group [35].

Health department sponsored CHW programs are not without their challenges. For instance, the Hawaii Department of Health partnered with federally qualified health centers (FQHCs) and the Hawaii Primary Care Association to create a CHW-led health promotion effort supporting diabetes prevention and hypertension control [36]. In this effort, CHWs were employed by the FQHCs. However, there were barriers in establishing links between the clinical and community settings, and sustainability was impacted by reimbursement [36]. The type of care that CHWs provide varies in effectiveness as well. For instance, in a pilot project for Texas’ Transformation and Quality Improvement Program, the Texas Department of Health and Human Services certified CHWs to work with patients with diabetes [37]. Blood sugar control was more rapid in patients who received in-person care from CHWs compared to patients who participated in telehealth with CHWs [37].

#### Federal Health Departments

At the national level, Alaskan Community Health Aides/Practitioners (CHA/Ps) operate under a scope of practice defined by the Indian Health Service, which is under the U.S. Department of Health and Human Services (HHS) [38]. CHA/Ps’ responsibilities include but are not limited to prenatal care and education, emergency deliveries, and general health education for teenagers. Many Alaskan communities are in rural and remote areas, and CHA/Ps are often the only healthcare providers for these communities [38].

HHS also runs a program called Healthy Start, which aims to address inequities in maternal and infant health [39]. To fulfill this mission, the program heavily relies on CHWs. A survey of Healthy Start programs revealed that 91% employed CHWs in some capacity. The CHWs work with women, infants, and their families, providing culturally competent services and addressing social determinants of health [39].

Finally, the U.S. Department of Veterans Affairs (VA) implemented a CHW program in 2002 called the Tribal Veterans Representatives (TVRs) [40]. TVRs, who are often American Indian and Alaska Native veterans themselves, help American Indian and Alaska Native veterans connect with the VA and gain access to its healthcare services by conducting outreach at veterans’ homes and community events. The program has trained over 800 TVRs and has led to multiple advances in veterans’ healthcare access, including the establishment of a primary care clinic and a resource center [40]. As of 2017, the program was still ongoing.

## Discussion

Health departments at all levels employ CHWs, from short-term interventions to long-term programs. They often partner with universities and community organizations to develop CHW programs and employ them. Health department involvement ranges widely, from providing only funding to organizations, to directly employing the CHWs involved in the interventions or programs. Many CHW programs focus on communities of color and low-income or rural communities. CHWs under federal health departments, in particular, often serve communities in the most remote areas of the country. Most programs have been successful, with common outcomes including improved health outcomes, lower medical costs, and decreased acute care utilization. Despite the successes of the programs, sustainability is unknown as it is unclear whether such programs continued after study publication.

State health departments have an additional layer of involvement in CHW workforce development through establishing certification and training standards for CHWs. Certification was consistently the biggest need identified by both CHWs and their employers. Some states such as Texas already have certification standards for CHWs, some are in the process of developing certification processes, and others have no certification processes at all. While most CHWs expressed interest in certification, cost was a primary concern, with many CHWs (and their employers) possibly unable to afford the certification process.

This review has several limitations. Our search of the literature likely did not identify every health department with a CHW program. We were only able to find CHW census surveys for a few states and the CHW workforce demographics from these states may not be broadly applicable to others. Furthermore, although there was a variety of study designs among the included articles, eight of the 29 articles were case studies. Further studies with stronger study designs, such as randomized controlled trials, would better support the effectiveness of CHW programs. Finally, information about state level CHW certification and reimbursement policies can change rapidly.

Most of the articles in our review were published before 2020. At the time of this writing, few articles have examined how specific health departments have employed CHWs during the COVID-19 pandemic. CHWs have played a major role in pandemic responses worldwide by raising awareness in their communities, participating in disease surveillance, and providing resources for preventative care [41], In the U.S., CHWs do not regularly respond to infectious disease outbreaks [41]. With the COVID-19 pandemic, however, the role of CHWs expanded dramatically. The White House’s American Rescue Plan invested heavily in the CHW workforce, providing funds for local and national organizations to hire, train, and deploy CHWs [42]. In September 2022, the White House announced that $225 million from the American Rescue Plan would go towards training over 13,000 CHWs, not just to support the U.S. pandemic response but also the public health workforce beyond the pandemic [42]. With the ending of the public health emergency, future studies should examine the impact of CHWs on community health outcomes post-pandemic. Future studies should also examine how CHWs are integrated within the focus of health departments and their scope of practice within governmental public health.

## Conclusion

Health departments are becoming increasingly involved in the CHW workforce, from developing CHW interventions and programs to establishing certification processes. Overall, CHW programs under health departments have made progress toward improving the health of marginalized communities. The results should encourage further investment in CHW initiatives and workforce development by governmental public health agencies.
